# An Outlook on Harnessing Technological Innovative Competence in Sustainably Transforming African Agriculture

**DOI:** 10.1002/gch2.202300033

**Published:** 2023-08-23

**Authors:** Senorpe Asem ‐ Hiablie, Daniel Dooyum Uyeh, Adesoji Adelaja, Kifle Gebremedhin, Ajit Srivastava, Klein Ileleji, Margaret Gitau, Yushin Ha, Tusan Park

**Affiliations:** ^1^ Biotechnology Department Shell International Exploration and Production Inc. Shell Technology Center Houston TX 77082 USA; ^2^ Department of Biosystems and Agricultural Engineering Michigan State University East Lansing MI 48824 USA; ^3^ Department of Agricultural, Food, and Resource Economics Michigan State University East Lansing MI 48824 USA; ^4^ Department of Biological and Environmental Engineering Cornell University Ithaca NY 14850 USA; ^5^ Department of Agricultural and Biological Engineering Purdue University West Lafayette IN 47907 USA; ^6^ Department of Bio‐Industrial Machinery Engineering Kyungpook National University Daegu 41566 Republic of Korea

**Keywords:** agricultural productivity and food security, digital technologies, indigenous knowledge, pandemic, resilience, system shocks

## Abstract

Agricultural value chains worldwide provide essential support to livelihoods, ecosystem services, and the growing bioeconomy. The coronavirus disease 2019 (COVID‐19) pandemic slowed down or reversed decades of agricultural growth and exposed the vulnerabilities of farmers and the food insecure in Africa, thus reiterating the need to build resilience, agility, and adaptability for a sustainable agriculture. Existing social, political, environmental, and economic challenges demonstrate that a path to faster sustainable growth is increased productivity through improved input quality, of which technical inputs are a part. This work presents a perspective calling for African innovative competence in technological and methodological applications and solutions as part of the most critical area of a holistic organization for social progress. It finds that while performances of previous agricultural transformation efforts offer insights for future directions, novel pathways fitting to the diversity of situations and contexts on the continent are needed. These may include vertical agriculture in land‐constrained regions to grow high‐value products, ocean or sea farming in coastal regions, development of multiple‐harvesting crops, and self‐replicating plants. Developing standards that integrate current scientific methodologies and technologies with indigenous knowledge for agricultural growth and disaster management will bring the complementary benefits of both worlds into optimal development.

## Graphical abstract

Agricultural value chains globally support livelihoods, ecosystems, and the bioeconomy. The COVID‐19 pandemic exposed vulnerabilities in African agriculture, emphasizing the need for resilience and adaptability. To achieve sustainable growth, Africa should focus on innovative technological solutions like vertical agriculture, ocean farming, and multiple‐harvesting crops while integrating scientific and indigenous knowledge for optimal development.

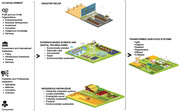

## 1 Vulnerabilities of African Agriculture

With strong domestic and cross‐border linkages, agriculture and related value chains worldwide enhance food security and provide essential support to livelihoods, ecosystem services, and increasingly, feedstocks for the growing bioeconomy. Shortly after the centennial of the 1918 H1N1 global influenza pandemic, which infected an estimated 500 million people globally and killed ≈2 percent of Africa's population, the 2019 coronavirus disease (COVID‐19) pandemic caused by the SARS COV 2 virus raised its ugly head. As predicted by epidemiological investigations of the 1918 pandemic, although advances have been made in countermeasures such as improved diagnostics, vaccines, community mitigation efforts, and communications, remaining gaps in disease management systems made the world's pandemic response readiness inadequate for another event of similar or larger magnitude.^[^ ^1^ ^]^ Both pandemics caused major disruptions in agricultural production and related activities globally and led to significant breakdowns in supply chains, with adverse effects on food supply and security in Africa.^[^ ^2^, ^3^ ^]^ This further highlights the need to transform current agrifood systems, as well as pandemic response and healthcare systems.

Coming on the heels of other shocks such as conflicts, macro‐economic downturns, and climate‐related disasters, the COVID‐19 pandemic exacerbated the problems facing agriculture and aggravated the food insecurity situation in much of Africa.^[^ ^3^ ^]^ However, it provided an opportunity to develop a holistic understanding of how a resilient agrifood system should function. A holistic and resilient agrifood system encompasses a complete value‐chain, including production, processing and storage, and distribution of the final product to the consumer. This also includes access to inputs for production and services, as well as safety and well‐being of the workforce involved in production. Most countries in the world, including those in Africa, were not prepared for disruptions that occurred in the entire food value chain. Even when food was available at the farm‐level, lockdowns and curfews prevented movement of goods, services, and people. Farmers could not take their goods to the market to sell, and supermarkets were opened for limited hours. Huge post‐harvest losses were incurred by farmers as highly perishable fresh produce that could not be readily sold locally or exported due to the temporary closure of borders was wasted.^[^ ^4^, ^5^ ^]^ Food processing factories were closed due to worker illness from the COVID‐19 disease resulting in disruption in food supply.

Some of the problems faced in African countries with respect to food access during the height of the pandemic were due to a lack of existing coordination by various agencies of the government and the private sector, especially with regards to curfews and restricted movements placed on the populace. For example, better coordination between the transport sector and agriculture could have prevented post‐harvest losses and lack of access, which were encountered during the pandemic. The lessons learned from these disruptions should provide private, government, not‐for‐profit, and international organizations with better understanding of how to design agrifood systems for resilience against unanticipated events. Additionally, the pandemic showed the world that we are interdependent and need to be concerned about events that occur beyond our borders. Geopolitical events such as the war between Russia and Ukraine, coming later in the pandemic, now pose another food security threat to some African countries that depend on Ukrainian grain imports. The African continent must build food self‐sufficiency and reduce dependency on food imports.

Evidence suggests that due to its resilience, agriculture can bounce back from minor shocks and stressors such as conflicts, droughts, and floods. For example, it has been shown that natural disasters have not had long‐term adverse effects on agricultural growth and economic transformation, hinting at the resilience of agriculture.^[^ ^6^ ^]^ It remains to be seen how lasting the damage caused by COVID‐19 to agriculture and related value chains would be. Given how prolonged, far‐reaching, and virulent it has been, the long‐term transformational effects of COVID‐19 and its ability to retard past gains and recoveries should be of major concern.

## 2 Africa and Global Food Security

The Food and Agricultural Organization of the United Nations (FAO) estimated that 60% of the world's uncultivated arable land is on the African continent.^[^ ^7^ ^]^ Possessing diverse agro‐ecological and climatic zones (from tropical rain forests to dry and arid), a wide variety of crops and commodities can be produced on the continent.^[^ ^8^ ^]^ This presents opportunities for feeding growing populations in Africa and the world if existing challenges such as low technology input, policy and human capital deficiencies, and adaptation to and mitigation of the impacts of climate change, are addressed. In its latest report on the state of the world's food security and nutrition, the FAO noted the slow progress being made toward set targets for reducing hunger, malnutrition, and food insecurity worldwide.^[^ ^9^ ^]^ Major challengers to the attainment of global food security and nutrition identified include geo‐political destabilization, socio‐economic shocks, and high costs of nutritious food. Added to these challenges are projected increases in temperatures and extreme weather events, changes in precipitation patterns, and reductions in water availability resulting from global warming. Transforming agrifood systems sustainably and inclusively will help attain resiliency in the African food value chain while helping to promote global food security and nutrition.

Most Sub‐Saharan African countries (SSA) had typically experienced relatively slower agricultural growth than the rest of the world until the year 2000. From 2000 to 2018, the rate of farm production growth in SSA roughly doubled (compared to the previous three decades), attaining close to 4%/year growth rate.^[^ ^10^, ^11^, ^12^ ^]^ While this made SSA the fastest growing region of the world, this growth was not sufficient to close the widening gap between food supply and demand to address growing food insecurity challenges.

**Figure 1:**
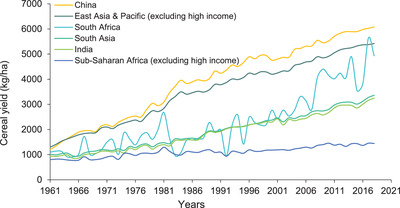
Sources of growth output for cereal yield in Sub‐Saharan Africa and other world regions.^[^ ^10^, ^20^ ^]^

**Figure 2:**
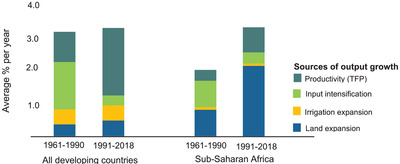
Sources of agricultural output growth in Sub‐Saharan Africa and developing countries.^[^ ^11^, ^21^ ^]^

Recent growth in agricultural production, albeit insufficient, came largely as a result of land expansion, not labor productivity (economic output per labor hour) growth^[^ ^11^ ^]^ (**Figures** **1** and **2**) as compared to productivity‐led growth in many other regions of the world. Rapid urbanization and diet transformation due to recently achieved economic growth is creating a new set of challenges for African agrifood systems that must be addressed. The proportion of Africans living in urban areas is projected to increase to above 60% by 2030. This will potentially lead to decreases in the labor force available for production agriculture and increasing demand for imported foods due to changing diets linked to urban lifestyles.^[^ ^13^, ^14^ ^]^ Agricultural productivity‐led growth is critical to the process of economic transformation. Such growth is important, given the large proportion of Africans living in poverty and who rely on agriculture for their livelihoods (Figure 1).

The impact of growth in irrigation expansion on agricultural productivity has historically remained fairly flat in SSA and most developing countries (Figure 2). Globally, the majority (70%) of the freshwater withdrawal has been for agricultural use.^[^ ^15^ ^]^ The FAO estimated the available internal renewable freshwater resource in SSA as 3 884 000 m^3^ in 2015. Renewable internal freshwater resources flow refers to internal renewable resources (internal river flows and groundwater from rainfall) within a country or region.^[^ ^16^ ^]^ The majority of the countries in SSA have been characterized as having no water stress (withdrawals <25% of total resources), with the exception of Kenya and Ethiopia, which have low stress (withdrawals 25% – 50% of total resources) and Sudan which is critically stressed (withdrawals >100% of total resources). However, following a global trend in declines of renewable freshwater sources per capita due to population growth, SSA has seen a decrease from 18 000 m^3^ in 1961 to 4 000 m^3^ in 2019 per capita, according to the FAO.^[^ ^16^ ^]^ It should also be noted that lower irrigation water use may be related to underdevelopment of the irrigation sector.

Whether in the form of freshwater withdrawal or rainfed sources, water is usually an essential input for agriculture. Sustainable levels of water resources are attained when the rates of withdrawal or loss are below replenishment. Evapotranspiration is the combined process of water loss through evaporation from the land's surface and transpiration from plants. As ET is a crucial process in the hydrological cycle which influences the availability and distribution of water resources in various ecosystems as well as regional and global climate patterns, understanding evapotranspiration (ET) patterns and dynamics in SSA would be essential for sustainable agriculture, water management, and proper ecosystem functioning. Studies have shown spatial and temporal variability in evapotranspiration across Africa. Vegetation type, including forests, grasslands, and savannahs, plays a critical role in regulating ET patterns across different ecosystems. Therefore, anthropogenic activities that cause changes in land use and vegetation cover, such as agricultural expansion, can have impacts on ET processes by altering the surface energy balance, resulting in modified evapotranspiration rates. Advances in remote sensing technologies to help assess the impacts of land use and land cover changes on evapotranspiration integrated with ground‐based modeling techniques can help contribute to a better understanding of ET patterns across the continent.^[^ ^17^, ^18^, ^19^ ^]^

Although the majority of the labor force in SSA (up to 70% in some countries) is employed in the agricultural sector, agriculture contributes only an average of 15% to the continent's gross domestic product, ranging from 2.4% to 53% in some countries.^[^ ^22^ ^]^ The process of economic transformation requires that the benefits of labor productivity growth in agriculture be leveraged into overall economic growth^9^. Economic transformation requires the transition of highly skilled workers from agriculture to other sectors such as industry and services, which generate greater value added. From the perspective of food security and livelihoods, the broader agri‐food system is a natural temporary and permanent destination for highly skilled agricultural workers. So while productivity growth in agriculture is central to economic transformation in SSA, the region has lagged behind other regions.

## 3 Productivity Growth is Critical to Food Security and Effective Economic Transformation

**Figure 3:**
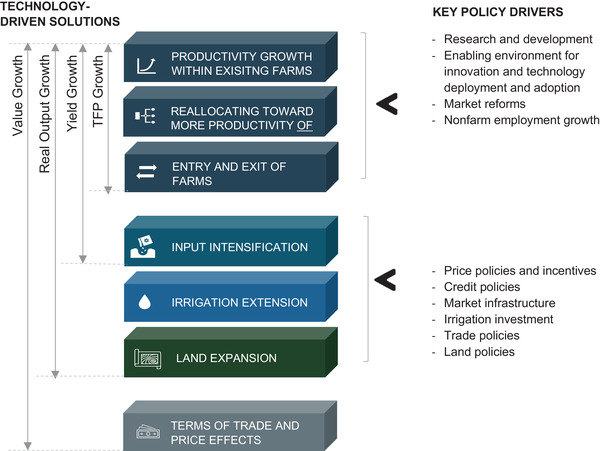
Sources of agricultural growth output.^[^ ^24^ ^]^ Increased Total Factor Productivity (TFP) is a result of the adoption of new technologies by existing farms and increased efficient resource use by farms (including the expansion of the most productive farms).

Barriers to productivity growth in agriculture range from social, political, governance, cultural and environmental factors to economic factors. However, perhaps the most critical reasons for low productivity in SSA agriculture are the generally low levels of technological inputs and the low usage of standard methodologies. On average, ≈70% of farm work is performed manually, 20% by draft animals and only 10% by mechanical power.^[^ ^23^ ^]^ The path to faster growth is increased productivity through improved input quality of which technical inputs are a major part (**Figure** **3**).^[^ ^24^ ^]^ This calls for African innovative competence in technological and methodological applications and solutions as part of the most critical area of a holistic support infrastructure for social progress. Transforming agriculture on the African continent into an efficient, productive, agile, and resilient sector will provide opportunities for enhancing food security, stimulating economic growth, and improving quality of life sustainably, equitably, and responsibly.

Currently, Africa imports food products equal to a third of calories consumed on the continent, and it is estimated that food imports will increase to $110 billion by 2025.^[^ ^23^ ^]^ This makes the continent vulnerable to supply chain disruptions as observed in the recent and previous pandemics. Non‐farming activities account for only 38% of the agricultural value chain in Africa, compared to 78% globally, an indication of the potential for significant growth in the agribusiness sector.^[^ ^13^ ^]^ This highlights the need for building regional capacity to grow strategically selected crops thus protecting against supply chain disruptions. In fact, it has been reported that an investment of between $ 315 bn and $ 400 billion in transforming 18 agricultural value chains over a ten‐year period will open up markets worth $80 billion per annum for Africa.^[^ ^4^ ^]^

Technology adoption has been largely known to lead to increasing economic returns, with examples in Africa (Tanzania and Ethiopia, for example).^[^ ^25^, ^26^, ^27^, ^28^ ^]^ Globally, the use of genetically modified technology in agriculture at the farm level has been shown to positively impact the net economic benefits from the cultivation of soybeans, corn, cotton, and canola. This amounted to $186.1 billion between 1996 and 2016.^[^ ^29^ ^]^ The economic gains were split nearly evenly between farmers in developed countries (52%) and their counterparts in developing countries (48%). The majority of the gains (65%) were due to yield and production increases, while the remainder (35%) was associated with cost savings.

The impact of increasing technology on farm profitability is dependent on various contextual factors, including local agroecological region, farming system type, farm size, input costs, market conditions, and the specific technology being adopted.^[^ ^30^, ^31^, ^32^ ^]^ Profitability outcomes are also determined by the level of technology adoption, farmers' skills and capacity to effectively use and manage the technology, access to information and training, and supporting institutional strategies.^[^ ^33^ ^]^ It is also important to note that technological advancements alone may not guarantee increased profitability if farmers lack the necessary complementary inputs, infrastructure, or market linkages to fully exploit the technology's benefits.^[^ ^33^ ^]^ Access to credit, reliable input supply chains, and market integration are also essential for translating technology adoption into improved profitability.^[^ ^32^ ^]^ Factors such as socioeconomics and labor availability can also shape interact with farm technology adoption.^[^ ^34^ ^]^ A comprehensive understanding of the local context is therefore essential in evaluating the potential gains.

## 4 Learning from Success Stories

The pathways and performances of previous agricultural transformation efforts offer insights for future directions. Agriculture in industrialized nations underwent steady growth in scale and speed using mechanical innovations during the 20^th^ century. An example of a recent fast agricultural transformation is in The Republic of Korea (South Korea), where layout architecture, standardization and workforce development were central to the growth of mechanization. The war‐ravaged South Korea, with low initial levels of agricultural productivity and a large food insecure population in the early 1950s, was able to attain a 98.6% adoption of mechanization for rice production and processing and 61.9% for dryland field crops in less than a century.^[^ ^35^ ^]^ Central to this was establishing the Korea Agricultural Machinery Industry Cooperative (KAMICO) by the Korean Agricultural Machinery and Equipment Manufacturers and the redesign and swapping of lands.

A key task assigned to KAMICO was standardization. This provided synchronization and ensured that machinery manufacturers found the right components from parts manufacturers. Cross‐cutting effects such as quality assurance, durability information, and necessary spare parts for repairs and replacements during an equipment's lifespan ensured improved return on investment. These improved returns were crucial for sustained growth as farmers could buy new types of equipment to mechanize other aspects of their farms. Agricultural education in tertiary institutions were also established during this period.

The redesigning of farm layout was essential for mechanization as it allowed easy access and movement of farm machinery between farms and made what would ordinarily be disparate small‐scale farms to become of commercial size. This was enabled by government policies that allowed farmers to easily swap irregularly‐shaped portions of their farmlands with others in order to attain shapes that were operable by farm machinery such as combines. These benefits had other cross‐cutting effects such as cost‐effective connected irrigation systems across farms which helped the country expand its irrigation drive. Another benefit was flood control from the connected drainage systems. This land reform is benefiting the recent growth of robotics where path planning is critical.

In countries such as the United States, having developed effective growth in mechanization in the early 20^th^ century, which had leveled off by the 1960's, there was a move toward the adoption of approaches based on chemical and biological sciences and improved management practices.^[^ ^36^ ^]^ Plant breeding and new crop strains, improved nutrients (fertilizer), and more readily available water input, along with pest and disease management in some world regions, contributed to increased crop (particularly cereal) yields during the Green Revolution.^[^ ^37^ ^]^ Another example of productivity‐led growth in Asia is Bangladesh, where the adoption of new technologies and innovations (low‐cost groundwater irrigation, aquaculture techniques, and improved crop varieties), macro‐economic reforms (diversification of the agricultural economy for example), and market liberalization drove rapid change in agricultural and economic development. One of the main outcomes was a 90% reduction in the need for emergency food assistance between 2008 and 2016 compared to the 1990s.^[^ ^10^, ^11^, ^21^ ^]^

Examples of agricultural productivity‐led growth could be found in Ethiopia and Ghana. Emerging from a devastating famine due to conflicts and severe droughts in the latter part of the 20^th^ century, Ethiopia achieved one of the fastest economic growth rates in the world. Agricultural output more than tripled over 25 years with an average growth of 5%/year between 1993 and 2013. Ethiopia did this by placing emphasis on raising the productivity of smallholder farmers.^[^ ^11^ ^]^ In Ghana, agriculture's total factor productivity growth (TFP: total inputs of land, labor, and capital inputs combined) contributed to an average economic growth of 5% per year.^[^ ^10^ ^]^ In fact, Ghana's gross agricultural output grew at nearly the same rate as the rest of the economy, each year since 1983. In addition to the total factor productivity, which accounted for nearly all of the country's growth in agricultural output, diversification of agricultural products played a major role as well.^[^ ^11^, ^38^ ^]^

## 5 The Way Forward with Technology – Calls for African Innovative Competence

Despite the immense merits of agricultural industrialization and intensification, concerns about the negative environmental impacts and resource constraints further confounded by conventional agriculture's impact on and vulnerability to climate change calls for more innovative approaches. Certain approaches for attaining agricultural productivity improvement have been criticized for having drawbacks in some regions in the past. Despite its achievements, the Green Revolution for example, has been criticized for negative impacts including human health impacts and environmental degradation (including soil erosion, water pollution, and loss of biodiversity due to intensive use of chemical fertilizers and pesticides) as well as groundwater resources depletion resulting from the reliance of high‐yielding varieties on irrigation.^[^ ^39^, ^40^, ^41^, ^42^ ^]^ The focus on high‐yielding varieties have also been blamed for the neglect and loss of traditional crop diversity and consequently, reducing resilience to pests, diseases, and changing climatic conditions.^[^ ^39^ ^]^ Furthermore, socio‐economic inequalities as a result of benefits being concentrated among large‐scale farmers were seen to marginalize smallholder farmers and further exacerbate income disparities.^[^ ^41^ ^]^ Loss of indigenous knowledge and cultural practices due to the introduction of modern agriculture that displaced indigenous crops and traditional farming systems have also been seen as negative impacts.^[^ ^40^ ^]^ These undesirable outcomes associated with past efforts to increase agricultural productivity could serve as lessons for future endeavors in order to help minimize negative impacts and well manage tradeoffs. This can be done by seeking more sustainable production systems and enhancing environmental services from agriculture, for example.^[^ ^41^ ^]^

The need for more sustainable production systems ushered in the era of major interests in climate‐smart agriculture, an integrated approach focusing on increased productivity, enhanced resilience, and reduced greenhouse gas emissions^[^ ^43^ ^]^ often buoyed by digital innovations such as image sensors and geographic information systems, which usually generate big data. This integrated growth and sustainability framework is seemingly appropriate for meeting the food security challenges of African nations.

The COVID‐19 pandemic has exposed the vulnerabilities of farmers and the population to food insecurity in Africa, and has reiterated the need to build resilience, agility, and adaptability for sustainable agriculture. The solutions obviously will vary by region given the diversity in practices and resources. To transform African agriculture in a major way, novel pathways of production and post‐production must be imagined that are fitting to the diversity of situations and contexts. These may include vertical agriculture in land‐constrained regions to grow high value horticultural crops, or livestock housing systems; ocean or sea farming in coastal regions (>30 African countries have these) of seaweed for example for food, livestock feed, and energy; and development of perennial or multiple‐harvesting crops to take advantage for the long growing seasons, and self‐replicating plants. These could complement existing sustainable production systems.

In addition to increasing the level of mechanization that is currently very low in Africa, greater connectivity between farms would be essential in building resilience and agility.^[^ ^44^ ^]^ Farm labor in some parts of the African continent is sourced locally while the farm managers and owners live away from the farms. With the limitations in movement imposed to mitigate the spreading of viruses during pandemics, remote monitoring, decision‐making, and management would be essential. Applications of intelligent, low‐power‐consuming interconnected sensing and robust communication systems offer means for efficient monitoring and control of farms using Internet of Things (IoT) and Internet of Everything (IoE) remotely. For example, irrigation technologies are becoming more popular in Africa, and installing actuators would enable remote control of these systems using the IoT. The IoT and IoE also provide other opportunities such as aggregating much needed data for allowing a shift from generalized management to highly optimized, individualized, real‐time and data‐driven, continuous monitoring of crops, water, and livestock. Data analytics for process optimization, precise crop nutrient application, irrigation, and pest and disease control, precise livestock feeding and medication, and prediction of machine and building failures are other ways agriculture can be transformed within the next decade.^[^ ^45^ ^]^

The backbone of digital agriculture is infrastructure for connectivity, power availability, technological know‐how, and access to the market. The growth of telecommunications networks and electrification on the African continent in the past decade has been trending upward and so have professionals in those sectors. The percentage of the population in lower‐middle‐income Sub‐Saharan African countries that use the internet increased from <1% to over 30% between the early 2000s and 2018.^[^ ^11^ ^]^ By 2019, ≈80% of sub‐Saharan Africans owned mobile cellphone subscriptions. Likewise, an increase was recorded in the percentage of rural sub‐Saharan Africans with access to electricity. This rose from 9% to 31% between 2000 and 2017.^[^ ^6^ ^]^ The rise of pioneering innovative financial technology solutions from the continent is evidence of these developments. Current efforts are underway on the continent to develop and expand the use of high bandwidth connectivity technologies such as 5G, Low‐earth orbit (LEO) satellites, and Low power wide area network (LPWAN).

## 6 Leveraging Indigenous Knowledge

In developing technologies, methodologies, and human capital, it is important to leverage the wealth of diversity of indigenous knowledge that abounds on the continent.^[^ ^46^, ^47^ ^]^ This would also help boost quicker assimilation through familiarity, inclusivity, and social acceptance. Indigenous knowledge refers to the local knowledge or capacity (often technological, social, economic and philosophical, learning system, and governance) that is unique to a given culture and acquired by local people through the accrual of experiences, informal experiments, and close understanding of their environment.^[^ ^48^, ^49^, ^50^ ^]^ Knowledge of the functioning of complex ecosystems and techniques for managing natural resources are gained from centuries of living in and interacting closely with these systems.^[^ ^48^ ^]^ Many examples of indigenous knowledge used for disaster management have been documented.^[^ ^51^, ^52^, ^53^ ^]^ Records also exist of the use of indigenous knowledge in food production.^[^ ^54^ ^]^ For example, a 700‐year‐old indigenous regenerative soil management system that is currently practiced in West Africa in which targeted waste disposal transformed highly weathered low‐quality tropical soils into fertile, carbon‐rich soils that contributed nearly a quarter of a farm household's income albeit covering a relatively small area while also offering a carbon sequestration solution has been described.^[^ ^55^ ^]^

**Figure 4:**
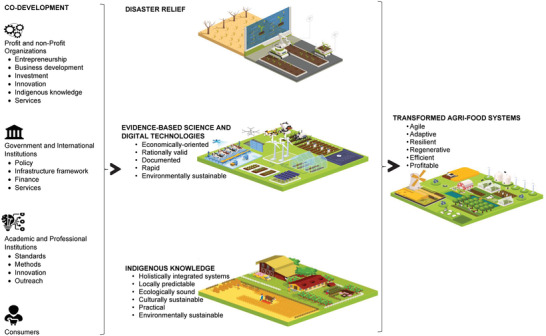
Schematic showing the integration of technologies and methodologies for transforming Africa agri‐food systems.

Integrating current digital agricultural technologies and methodologies with indigenous knowledge will bring the complementary benefits of both worlds into optimally developing an African agriculture that is resilient, agile, adaptable, and regenerative, thus sustainable. Indigenous knowledge offers a holistic, integrated systems approach that is locally predictable and relevant, ecologically and culturally sustainable, and practical (**Figure** **4**). When using indigenous knowledge, intellectual property concerns would need to be considered as well. Technologies and methodologies based on scientific evidence have the benefits of rational validity with predictability founded on basic natural principles as well as documentation, economic sustainability, and relatively rapid acquisition.^[^ ^48^ ^]^ They are often standardized as well.

## 7 Human Capital Development, Land Reform, and Sustainable Outreach Programs

In addition to the necessity of technological and methodological modernization, other equally critical measures that need to be implemented and integrated in a holistic approach of development are human technical and financial capacity. Human technical capacity can be built through training farmers and growers using sustained extension and outreach programs (with translations into local languages) and curriculum changes that prepare youth to work on and own commercial farms.^[^ ^56^, ^57^, ^58^ ^]^ Technological innovations should be coupled with innovative modes of transferring know‐how to farmers via all forms of digital tools, which can be delivered at scale and at low cost. Governments should be open to equitable land reform and incentivize farmers to organize in communal farming arrangements that are suitable for mechanization and irrigation. Farmers should have access to the market to sell their produce through crop/grain stock markets and get services. Modern information technology has improved the capability of communities across the continent to access and share information, experiences, and practices. It is expected that innovations considered should foster environmental protection, economic growth, and human‐capital empowerment, especially for women and youth.^[^ ^59^ ^]^ Youth are the future of any society and their involvement is critical to the transformation of agri‐food systems. The integration of modern technologies into agriculture will help make the sector more appealing to the youth in SSA who, in general, consider agriculture to be archaic.

With indigenous knowledge, practical skills, entrepreneurial mindset, and resourcefulness, skilled African farmers possess the capability to adopt technology and effectively incorporate it into their decision‐making processes with the support of peer‐to‐peer knowledge sharing and extension services. Having the domain experience and expertise in managing local agroecosystems, crop varieties, and traditional farming practices, make farmers holders of knowledge repositories about local production contexts and practices thereby making them key sources of innovation.^[^ ^46^ ^]^ This is critical in bridging sources of knowledge and fostering open innovation.^[^ ^47^, ^60^, ^61^, ^62^, ^63^, ^64^, ^65^ ^]^ The domain knowledge will enable the farmers to make informed decisions when adopting and adapting technology to suit local conditions and contexts. African farmers have exhibited a willingness to experiment with new approaches and technologies as well as being resourceful and adaptive, employing creative problem‐solving techniques to address challenges in agriculture.^[^ ^46^, ^66^ ^]^ This entrepreneurial spirit fosters the adoption of technology and drives innovation in farming practices. Additionally, well‐designed extension services and capacity‐building programs that provide education, training, and advisory support, will enhance farmers' understanding of technology options and enable them to make informed decisions.^[^ ^25^, ^67^ ^]^ Furthermore, farmers in Africa actively participate in learning networks and engage in knowledge exchange activities.^[^ ^68^, ^69^, ^70^ ^]^ These networks, such as cooperatives, farmer field schools or community‐based organizations, provide opportunities for sharing experiences, best practices, and technical know‐how.^[^ ^71^ ^]^

## 8 Concluding Thoughts

The following key points can be highlighted from this paper:

1. To feed the current and future populations (for which growth has been predicted) while withstanding shocks such as pandemics, transformation in technology and methodology of agriculture and the food value chain in SSA must occur in a holistic manner focusing on youth, women, and small‐holder farmers. Human capital development, land reform, and sustainable outreach programs are integral components of the proposed holistic approach. The system must be profitable to be sustainable, contributing to larger economic growth.
2. Technology and methodology developed and deployed must be transformational in scope and scale. Novel pathways of production and postproduction befitting to the diversity of situations and contexts on the continent must be imagined. This may include vertical agriculture in land‐constrained regions to grow high value products, ocean or sea farming in coastal regions, and the development of perennial or multiple‐harvesting crops and self‐replicating plants.
3. Digital agricultural technologies, methodologies, and innovations must be integrated with indigenous knowledge to bring the complementary benefits of both worlds into optimally developing an African agriculture that is resilient, agile, adaptable, and regenerative.
4. Standardization of methodologies and technologies will help drive rapid deployment and scaling up.
5. To protect against future supply chain disruptions as seen during the COVID‐19 pandemic and similar shocks, it is necessary to build regional capacity to grow strategically selected food‐security crops incorporating indigenous knowledge and practices.
6. Governments should be open to equitable land reform and incentivize farmers to organize in communal farming arrangements that are suitable for mechanization and irrigation. Farmers should also have access to supporting services and markets to sell their produce.

In designing a resilient agri‐food system, logistics of the food supply chain from securing crops at the farm‐gate to storage facilities, food processing facilities, and food stores as well as distribution and retail centers need to be integrated to work together in order to deliver food to consumers at the least cost and carbon footprint. Stakeholders to help shift priorities toward a holistic approach may include country‐level government agencies, continent‐wide policy organizations, global development partners, research and education institutions, and the private sector. The design and implementation will require new ways of thinking and working which are driven by indigenous knowledge and guided by evidence‐based science and digital technologies. Factors impacting successful agricultural technology adoption do not always have the same effect within different systems. Rather, influencing variables depend on various factors including human specific influences (for example, farmer perception and societal acceptance), technology type, institutional type, and economic and market factors.^[^ ^31^, ^71^, ^72^, ^73^, ^74^ ^]^ Farm size, for example, has been shown to have mixed effects towards the adoption of technologies; sometimes positive, other times, negative.^[^ ^72^ ^]^ Understanding influencing factors of agricultural technologies are essential in attaining and sustaining success.^[^ ^75^ ^]^

## Conflict of Interest

The authors declare no conflict of interest
